# Supplementary material for: EnzML: multi-label prediction of enzyme classes using InterPro signatures
Source: BMC Bioinformatics. 2012 Apr 25;13:61. doi: 10.1186/1471-2105-13-61 (PMC3483700; doi:10.1186/1471-2105-13-61)
Supplement: Addtional file 5 — The Java code to format the data files, evaluate and predict. The file enzml_java_code.tar.gz contains the Java code used to format database data to ARFF and XML formats, to execute cross and train-test (jackknife) evaluations and to record evaluation results to database. More information is included in the readme.txt file and the Javadoc files. The code can be used with a MySQL database. To use a different database software, other JDBC drivers might be required. [file 1471-2105-13-61-S5.gz › java_code/enzml2011/doc/uk/ac/ed/inf/taweka/mulan/learn/ResultsSaver.html]

ResultsSaver


---


|  |  |  |
| --- | --- | --- |
| **Overview** \| **Package** \| **Class** \| **Use** \| **Tree** \| **Deprecated** \| **Index** \| **Help** | |  |
| **PREV CLASS**   **NEXT CLASS** | **FRAMES**    **NO FRAMES**     **All Classes** |  |
| SUMMARY: NESTED \| FIELD \| CONSTR \| METHOD | DETAIL: FIELD \| CONSTR \| METHOD |  |


---


## uk.ac.ed.inf.taweka.mulan.learn Class ResultsSaver

```
java.lang.Object
  uk.ac.ed.inf.taweka.mulan.learn.ResultsSaver
```

---

``` public class ResultsSaver extends java.lang.Object ```

Outputs cross-evaluation results to screen, file and database.

**Version:**
:   15 Aug 2010

**Author:**
:   Luna De Ferrari luna.deferrari-at-ed.ac.uk

---

**Constructor Summary**

`ResultsSaver(uk.ac.ed.inf.utils.database.DbManager experimentDbManager,
java.lang.String filePath)`


| **Method Summary** | |
| --- | --- |
| `java.lang.String` | `getFilePath()`             return the file path the results will be saved to |
| `boolean` | `getResultsCanBeSaved()` |
| `uk.ac.ed.inf.utils.database.DbManager` | `getResultsDbManager()` |
| `uk.ac.ed.inf.utils.database.TableManager` | `getTableManager()` |
| `java.lang.String` | `resultsString(uk.ac.ed.inf.utils.database.TableRow row)` |
| `int` | `saveResults(int runNumber, int trainArffId, int testArffId, java.lang.String startTime, java.lang.String endTime, MulanLearner learner, mulan.evaluation.Evaluation evaluationResults)` |

**Methods inherited from class java.lang.Object**

`equals, getClass, hashCode, notify, notifyAll, toString, wait, wait, wait`

**Constructor Detail**

### ResultsSaver

```
public ResultsSaver(uk.ac.ed.inf.utils.database.DbManager experimentDbManager,
                    java.lang.String filePath)
```


**Method Detail**

### getFilePath

```
public java.lang.String getFilePath()
```

:   return the file path the results will be saved to

---


### getResultsCanBeSaved

```
public boolean getResultsCanBeSaved()
```

---


### getResultsDbManager

```
public uk.ac.ed.inf.utils.database.DbManager getResultsDbManager()
```

---


### getTableManager

```
public uk.ac.ed.inf.utils.database.TableManager getTableManager()
```

---


### resultsString

```
public java.lang.String resultsString(uk.ac.ed.inf.utils.database.TableRow row)
```

---


### saveResults

```
public int saveResults(int runNumber,
                       int trainArffId,
                       int testArffId,
                       java.lang.String startTime,
                       java.lang.String endTime,
                       MulanLearner learner,
                       mulan.evaluation.Evaluation evaluationResults)
```


---


|  |  |  |
| --- | --- | --- |
| **Overview** \| **Package** \| **Class** \| **Use** \| **Tree** \| **Deprecated** \| **Index** \| **Help** | |  |
| **PREV CLASS**   **NEXT CLASS** | **FRAMES**    **NO FRAMES**     **All Classes** |  |
| SUMMARY: NESTED \| FIELD \| CONSTR \| METHOD | DETAIL: FIELD \| CONSTR \| METHOD |  |


---
